# A Retrospective Observational Study Comparing Short-Term Outcomes in Intracorporeal vs Extracorporeal Robotic Right Hemicolectomy

**DOI:** 10.7759/cureus.105788

**Published:** 2026-03-24

**Authors:** Gowtham Sundaram Venkatesan, Mohammed Arifuzaman, Vivek Peddakota, Sandeep Samson, Prem Thambi, Dharmendra Garg, Madan K Jha

**Affiliations:** 1 Colorectal Surgery, James Cook University Hospital, Middlesbrough, GBR; 2 General Surgery, James Cook University Hospital, Middlesbrough, GBR

**Keywords:** conventional right hemicolectomy, extracorporeal anastomosis, intracorporeal anastomosis, outcomes of robotic right hemicolectomy, right hemicolectomy, right-sided hemicolectomy, robotic surgery in oncology

## Abstract

Background

Robotic right hemicolectomy is increasingly performed for neoplastic disease, but the optimal method of anastomosis remains debated. Intracorporeal anastomosis (ICA) may offer advantages in reducing wound complications and postoperative ileus, while extracorporeal anastomosis (ECA) is technically simpler. Evidence specific to oncologic populations, however, is limited.

Methods

We conducted a retrospective observational study of patients undergoing robotic right hemicolectomy for neoplastic pathology at a tertiary academic centre between January 2023 and December 2024. Patients were stratified into ICA and ECA groups. Primary outcomes included early postoperative recovery (time to bowel movement, anastomotic leak, and 30-day readmission). Secondary outcomes assessed operative time, wound complications, postoperative ileus, systemic inflammatory response (C-reactive protein (CRP) and white cell count (WCC)), length of stay, and oncologic adequacy (lymph node harvest, tumour staging). Statistical analyses included t-test, Mann-Whitney U test, chi-square test, or Fisher’s exact test, with significance set at p <0.05.

Results

Eighty-three patients were included: 61 (73.5%) underwent ICA, and 22 (26.5%) underwent ECA. Baseline demographics, body mass index (BMI), and prior abdominal surgery rates were comparable between groups. ICA was associated with longer operative time (176.1 vs 153.7 minutes, p = 0.06). Anastomotic leaks were rare (ICA 2/61, 3.3%; ECA 0/22; p = 1.0). Wound infections occurred only in the ECA group (13.6% vs 0%, p = 0.02), and postoperative ileus was more frequent with ECA (13.6% vs 0%, p = 0.02). Readmission rates were similar (ICA 9.8% vs ECA 9.1%, p = 1.0). There were no significant differences in systemic inflammatory markers or length of hospital stay. Lymph node yield (24.9 vs 22.4, p = 0.33) and tumour stage distribution were comparable, confirming oncologic adequacy. One postoperative death occurred in the ICA group, unrelated to anastomotic integrity.

Conclusion

In robotic right hemicolectomy for neoplastic disease, ICA was associated with lower rates of wound infection and postoperative ileus compared with ECA, without increased leak rates or compromise in oncologic adequacy. Despite slightly longer operative times, ICA demonstrated favourable short-term morbidity. Prospective multicentre studies are warranted to confirm these findings and guide surgical decision-making in colorectal oncology.

## Introduction

Colorectal cancer remains one of the most prevalent malignancies globally, and right-sided colon cancers account for a significant proportion of these cases. Minimally invasive techniques, especially laparoscopic right hemicolectomy (LRH), have become the standard of care due to improved postoperative recovery, reduced pain, and comparable oncologic outcomes compared to open surgery [1,2].

During LRH, the creation of the ileocolic anastomosis can be performed either extracorporeally (ECA) or intracorporeally (ICA). The traditional ECA approach requires externalization of the bowel through an extraction incision, which can result in traction injury, restricted extraction site choice, and increased risk of wound complications [3,4]. In contrast, ICA allows an entirely intracorporeal reconstruction, minimizing bowel handling and mesenteric tension, which may translate into faster bowel recovery and fewer wound-related complications [5,6].

However, laparoscopic ICA requires advanced intracorporeal suturing and stapling skills, leading to a steep learning curve. The evolution of robotic-assisted platforms, with articulated instruments, tremor filtration, and superior 3D visualization, has mitigated these technical limitations [7,8]. Consequently, robotic right hemicolectomy (R-RHC) has been increasingly adopted, enabling precise dissection and a standardized ICA [9,10].

Several studies and meta-analyses have suggested that robotic ICA offers lower rates of wound infection and postoperative ileus compared to ECA, with no compromise in oncologic safety [11-17]. Nonetheless, the evidence remains limited by small sample sizes and heterogeneous study designs. Further, data specific to patients with neoplastic indications remain sparse.

The present study evaluates short-term postoperative outcomes of R-RHC performed with ICA versus ECA for neoplastic disease in a tertiary academic center. We hypothesize that ICA confers superior short-term outcomes without compromising oncologic adequacy.

## Materials and methods

Study design

This is a retrospective, observational study conducted at a tertiary care academic centre, analysing patients who underwent R-RHC between January 2023 and December 2024. The study specifically compares short-term postoperative outcomes between ICA and ECA techniques in patients operated on for neoplastic etiologies. The samples are categorized into ICA vs ECA. Six colorectal consultants with a special interest in robotic surgery have performed procedures, and the choice of approach was primarily the surgeon's preference.

Inclusion criteria were patients who underwent R-RHC with a final histopathological diagnosis of neoplastic disease (malignant or premalignant polyps) and who had complete perioperative and postoperative data available. Exclusion criteria were surgery performed for purely benign, non-neoplastic indications, incomplete records of operative details or postoperative outcomes, conversions to open surgery, or conversion to a laparoscopic approach. Data were extracted from institutional electronic health records and operative logs and compiled into a structured dataset. The variables that were analysed are listed in Table 1.

**Table 1:** List of variables analysed ECA, extracorporeal anastomosis; ICA, intracorporeal anastomosis; BMI, body mass index.

Major variables	Co-variables
Demographics	Age, gender, BMI, comorbidities
Operative details	Anastomotic type (ICA or ECA), surgical time, installation time, time in recovery, and surgical technique
Postoperative outcomes	Time to first flatus and stool, duration of hospital stay, readmission within 30 days (and reason), and complications within 30 days
Oncologic metrics	Lymph node harvest count, tumour staging (T status), and histopathology

Outcomes

The primary outcomes were time to first bowel movement (flatus or stool), anastomotic leak, and readmission within 30 days. Anastomotic leaks were identified based on one or more of the following: radiological evidence of contrast extravasation on CT imaging, perianastomotic fluid collection containing air or contrast, intraoperative confirmation during reoperation, clinical features consistent with leak (e.g., sepsis, peritonitis) requiring intervention. Postoperative ileus was diagnosed when two or more of the following were present on or after postoperative day 3, such as nausea or vomiting, inability to tolerate oral diet, abdominal distension, absence of flatus or stool, and radiological evidence of dilated bowel loops without mechanical obstruction.

Secondary outcomes included operative time, length of hospital stay, wound complications or delayed complications (up to 30 days), postoperative lymph node yield, and tumour stage for oncologic adequacy.

Statistical analysis

Statistical analyses were performed using SPSS Statistics (version 30; IBM Corp., Armonk, NY). Continuous variables were assessed for normality using the Shapiro-Wilk test. Normally distributed continuous data, such as operative time and lymph node yield, were compared using the Student’s t-test, whereas non-parametric variables, including recovery time, pain scores, C-reactive protein (CRP), and white cell count (WCC), were analysed using the Mann-Whitney U test. Categorical variables, such as anastomotic leak, wound infection, postoperative ileus, and history of previous abdominal surgery, were evaluated using the chi-square (χ²) test or Fisher’s exact test when expected cell counts were low. Test statistics (t, U, and χ² values) with corresponding p-values were reported for transparency. Statistical significance was defined as p <0.05.

For clarity, baseline demographic comparisons (age, BMI, and previous surgery) utilized Mann-Whitney U and chi-square tests. Operative outcomes (operative time, recovery time, pain scores, and lymph node yield) were analysed using t- or U-test depending on data distribution. Postoperative complications (anastomotic leak, wound infection, postoperative ileus) were assessed using Fisher’s exact test, while inflammatory markers (CRP and WCC on days 1 and 3) were compared using the Mann-Whitney U test.

## Results

A total of 83 patients who underwent R-RHC were included in the study. Of these, 61 patients (73.5%) received an ICA, while 22 patients (26.5%) underwent an ECA. The median age of the cohort was 73 years, with a range of 32 to 90 years. There were 39 male and 44 female patients, yielding a sex ratio of 0.89:1 (male-to-female). A history of previous abdominal surgery was noted in 49 patients (59%), while 34 patients (41%) had no prior surgical interventions. The median body mass index (BMI) was 28.22 kg/m², with a range from 16.96 to 42.41 kg/m², reflecting a wide variation in body habitus. The summary of demographic factors is listed in Table 2.

**Table 2:** Statistical comparison of baseline demographic characteristics between ICA and ECA groups ECA, extracorporeal anastomosis; ICA, intracorporeal anastomosis; BMI, body mass index.

Demographic factors	ECA	ICA	Statistical test used	Test statistics	p-Value
Total participants	22	61			
Age (years)					
Median age	70.5	74	Mann-Whitney U test	U = 652.0	0.92
Age range	60-85	32-90			
Sex					
Male count	7	32			
Female count	15	29			
Sex ratio	0.47:1	1.10:1	Chi-square (χ²) test	χ² = 2.56, df = 1	0.11
BMI					
Median BMI	26.18	28.49	Mann-Whitney U test	U = 608.5	0.22
BMI range	16.96-42.41	18.66-39.58			
Previous surgery	13	36	Chi-square (χ²) test	χ² = 0.00, df = 1	1

The median age in the ICA group was 74 years (range: 32-90), compared to 70.5 years (range: 60-85) in the ECA group. This difference was not statistically significant (p = 0.92; Mann-Whitney U test). There was no statistically significant difference in sex distribution between the ICA and ECA groups (p > 0.05; chi-square test). The median BMI was 28.49 kg/m² (range: 18.66-39.58) in the ICA group and 26.19 kg/m² (range: 16.96-42.41) in the ECA group. The difference in BMI between the groups was not statistically significant (p = 0.23; Mann-Whitney U test). Regarding surgical history, 36 patients (59%) in the ICA group and 13 patients (59%) in the ECA group had a history of previous abdominal surgery. There was no significant association between previous surgical history and the choice of anastomosis technique (p = 1; chi-square test). 

Outcome analysis

Table 3 shows that the ICA group demonstrated a longer mean operative time (176.13 ± 49.24 minutes) compared to the ECA group (153.68 ± 37.62 minutes), with this difference approaching statistical significance (p = 0.06). Pain scores on postoperative day 1 were higher in the ICA group (4.00 ± 2.29) than in the ECA group (2.00 ± 1.86), though the difference was not statistically significant (p = 0.20). On postoperative day 3, mean pain scores were nearly identical (2.00 ± 2.05 for ICA vs. 2.14 ± 2.27 for ECA; p = 0.93). The mean lymph node yield was 24.89 ± 11.07 in the ICA group and 22.41 ± 7.07 in the ECA group. This difference did not reach statistical significance (p = 0.33). Finally, six out of 61 patients (9.8%) in the ICA group and two out of 22 patients (9.1%) in the ECA group were readmitted within 30 days, showing no statistical difference between the groups (p = 1).

**Table 3:** Statistical comparison of key postoperative outcomes between ICA and ECA groups SD, standard deviation; ICA, intracorporeal anastomosis; ECA, extracorporeal anastomosis.

Outcome variable	ECA	ICA	Statistical test used	Test statistics	p-Value
Operating time in minutes					
Mean	153.68	176.13	Mann-Whitney U test	U = 589	0.06
SD	37.62	49.24			
Recovery time					
Mean	89.73	119.33	Mann-Whitney U test	U = 563	0.24
SD	38.11	80.41			
Pain score Day 1					
Mean	3.29	4.29	Mann-Whitney U test	U = 602.5	0.2
SD	3.35	3.03			
Pain score Day 3					
Mean	1.69	2	Mann-Whitney U test	U = 619.5	0.93
SD	2.02	2.52			
Lymph node yield					
Mean	22.41	24.89	Student’s t-test	t (81) = 0.98	0.33
SD	7.07	11.07			

Postoperative histology was reviewed for all patients, categorized into either malignancy (T-staged adenocarcinoma) or suspicious neoplastic lesions (including dysplastic and non-invasive lesions). The ICA group had a higher overall proportion of advanced-stage malignancies (T3 or greater: 39 ICA vs. 12 ECA), consistent with the higher number of malignant histologies in the ICA group. However, there was no significant difference in the histological patterns of suspicious neoplastic lesions between groups. The detailed histology results are listed in Table 4.

**Table 4:** Postop histology LG, low grade; TVA, tubulo-villous adenoma; HGD, high-grade dysplasia; Tis, tumour in situ; ICA, intracorporeal anastomosis; ECA, extracorporeal anastomosis.

Detailed post-op histology results	ECA	ICA
Hyperplastic polyp	1	0
LG TVA	0	6
LG TVA + HGD	1	1
Large sessile polyp LGD	0	1
Post-appendicular carcinoid excised with positive margin	0	1
Pseudo lipomatous changes	1	0
T staging for malignant histology		
T1	2	8
T2	4	4
T2 -- Polyp preop, but no cancer final histology	0	1
T3	7	26
T4		
T4a	2	10
T4a + 4b	1	0
T4a+4b	2	2
T4b	0	1
Tis	1	0

The overall incidence of anastomotic leak was low, observed only in the ICA group (2/61; 3.3%), and not in the ECA group (0/22), although this difference was not statistically significant (p = 1). There were no cases of intra-abdominal collection reported in either group. However, patients in the ECA group experienced significantly higher rates of both wound infection/dehiscence and postoperative ileus compared to the ICA group. Wound infection occurred in three out of 22 patients (13.6%) in the ECA group, whereas no such cases were noted in the ICA group (p = 0.02). Similarly, postoperative ileus was seen in three ECA cases (13.6%) and none of the ICA cases (p = 0.02). These findings suggest that ICA may be associated with a lower incidence of wound-related and functional postoperative complications compared to ECA. In the ICA group, a wider variety of complications was observed, including respiratory, gastrointestinal, thromboembolic, inflammatory, and urological events. The ECA group exhibited a single type of complication related to delirium. One mortality is observed in ICA. Postoperative outcomes comparing ECA vs ICA techniques are enumerated in Table 5.

**Table 5:** Statistical comparison of postoperative complications and inflammatory markers between ICA and ECA groups CRP, C-reactive protein; WCC, white cell count; IQR, interquartile range; ICA, intracorporeal anastomosis; ECA, extracorporeal anastomosis.

Postoperative complication	ECA	ICA		Statistical test used	Test statistics	p-Value
Anastomotic leak	2	0		Fisher’s exact test		1
Wound infection/dehiscence	0	3		Fisher’s exact test		0.02
Postoperative Ileus	0	3		Fisher’s exact test		0.02
CRP						
Day 1 -- Median (IQR)	78.00 (87.25)	75.00 (85.00)	Mann-Whitney U test		U = 640.5	0.75
Day 3 -- Median (IQR)	106.00 (100.00)	112.00 (112.00)	Mann-Whitney U test		U = 628.0	0.77
WCC						
Day 1 -- Median (IQR)	12.15 (2.58)	12.00 (4.70)	Mann-Whitney U test		U = 612.0	0.4
Day 3 -- Median (IQR)	9.05 (2.45)	9.10 (3.10)	Mann-Whitney U test		U = 598.5	0.81

Postoperative inflammatory response was assessed using CRP and WCC measurements on postoperative days 1 and 3 for both anastomotic techniques. The median CRP and WCC values were comparable between the ICA and ECA groups at both time points. Statistical comparison using the Mann-Whitney U test revealed no significant differences in CRP on day 1 (p = 0.75) or day 3 (p = 0.77), and no significant differences in WCC on day 1 (p = 0.40) or day 3 (p = 0.81). These results indicate that the early postoperative systemic inflammatory response was similar between the two groups.

Length of stay

**Figure 1:**
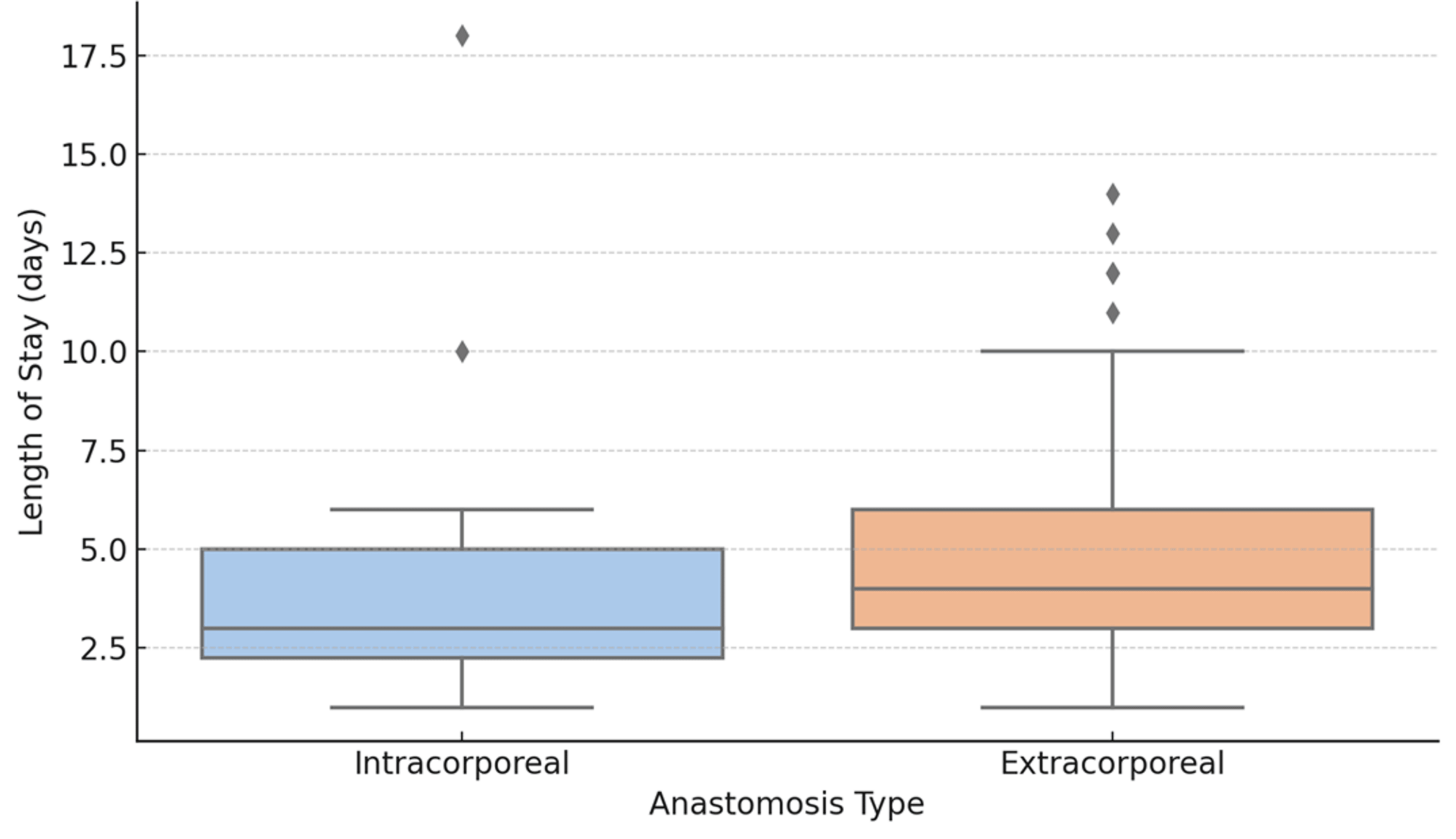
Shows the length of stay among ICA vs ECA in a box plot ICA, intracorporeal anastomosis; ECA, extracorporeal anastomosis.

The postoperative length of hospital stay was analysed for both ICA and ECA groups. One patient in the ICA group had a postoperative death; it was excluded from the statistical analysis to preserve the validity of the comparison. Among the remaining patients, the median length of stay was slightly different between the groups. However, the difference was not statistically significant (p = 0.14, U = 576, Mann-Whitney U test). Figure 1 shows a boxplot that was used to visualize the distribution, showing similar interquartile ranges and spread in both groups. These findings indicate that the choice of anastomotic technique did not significantly influence postoperative length of hospital stay in this cohort, after accounting for non-survivors.

## Discussion

This retrospective analysis compared ICA and ECA in R-RHC for neoplastic disease. The findings are consistent with growing evidence that ICA may enhance postoperative recovery without increasing complication rates [5]. The slightly longer operative time observed in the ICA group aligns with previous studies, which attribute this to the technical demands of intracorporeal suturing [6,12]. However, this additional time is offset by clinical benefits, including lower wound infection and postoperative ileus rates. Similar findings have been reported by Cleary et al. [6], Squillaro et al. [15], and Trastulli et al. [2], who demonstrated faster bowel recovery and reduced wound complications in robotic ICA compared with ECA.

The absence of wound infection and postoperative ileus in the ICA group may be explained by reduced bowel manipulation, less mesenteric traction, and smaller extraction incisions [14,15]. Furthermore, robotic articulation allows a tension-free, well-aligned anastomosis, potentially contributing to better functional outcomes. These physiological advantages have been validated in multicentre studies and meta-analyses showing lower rates of surgical-site infection and faster recovery of bowel function with ICA [16].

Anastomotic leak rates in this study were low and comparable between ICA and ECA, consistent with prior literature reporting leak rates under 5% in both techniques [16]. Importantly, no intra-abdominal collections were observed, reinforcing the technical reliability of both methods when performed by experienced colorectal surgeons. Although the length of hospital stay was not statistically different, a trend toward faster recovery in the ICA group mirrors previously reported data [16]. 

Oncologic outcomes, including lymph node yield and T-stage distribution, were comparable between groups, confirming that ICA does not compromise oncologic adequacy [16]. Indeed, improved specimen handling in ICA may facilitate more controlled mesocolic excision and reduce spillage risk [18]. Postoperative inflammatory markers (CRP and WCC) were comparable between groups, echoing findings from Zhang et al. [18] and ROLARR [19], which showed no significant difference in systemic inflammatory response between ICA and ECA techniques.

While these results are encouraging, the study’s retrospective design and limited sample size constrain definitive conclusions. Potential confounders, such as surgeon experience and case complexity, may have influenced outcomes. Nevertheless, the findings align with recent literature, including multicentre data and meta-analyses, underscoring that ICA provides equivalent oncologic outcomes with superior short-term morbidity profiles [16,18].

Future multicentre randomized studies, such as the ongoing MIRCAST and COLOR IV trials, are expected to validate these results and guide best practices [19]. Additionally, cost-effectiveness and long-term quality-of-life assessments are necessary to fully establish the role of robotic ICA in colorectal oncology.

## Conclusions

In this retrospective cohort study of patients undergoing R-RHC for neoplastic disease, ICA was associated with lower rates of wound-related complications and postoperative ileus compared to ECA. Despite a modestly longer operative time, ICA did not lead to increased systemic inflammation, anastomotic leak, or prolonged hospital stay. Oncologic adequacy, as assessed by lymph node harvest and pathological staging, was comparable between techniques.

These findings support the safety and potential short-term benefits of ICA in R-RHC, particularly in reducing early postoperative morbidity. While the results are promising, randomized controlled trials with larger cohorts are needed to validate these outcomes and further inform surgical decision-making in colorectal oncology.
